# Guy’s cancer cohort – real world evidence for cancer pathways

**DOI:** 10.1186/s12885-020-6667-0

**Published:** 2020-03-17

**Authors:** C. Moss, A. Haire, F. Cahill, D. Enting, S. Hughes, D. Smith, E. Sawyer, A. Davies, J. Zylstra, K. Haire, A. Rigg, M. Van Hemelrijck

**Affiliations:** 1 King’s College London, School of Cancer and Pharmaceutical Sciences, Translational Oncology and Urology Research (TOUR), Guy’s Hospital, 3rd Floor Bermondsey Wing, London, SE1 9RT UK; 2 Comprehensive Cancer Centre, Guy’s and St Thomas’ NHS Foundation Trust, London, UK; 3 Department of Upper Gastrointestinal Surgery, Guy’s and St Thomas’ NHS Foundation Trust, London, UK; 4 South East London (SEL) Accountable Cancer Network, Guy’s and St Thomas’ NHS Foundation Trust, London, UK

**Keywords:** Cancer, Observational data, Real world evidence

## Abstract

**Background:**

The burden of disease due to cancer remains substantial. Since the value of real-world evidence has also been recognised by regulatory agencies, we established a Research Ethics Committee (REC) approved research database for cancer patients (Reference: 18/NW/0297).

**Construction and content:**

Guy’s Cancer Cohort introduces the concept of opt-out consent processes for research in a subset of oncology patients diagnosed and treated at a large NHS Trust in the UK. From April 2016 until March 2017, 1388 eligible patients visited Guy’s and St Thomas’ NHS Foundation Trust (GSTT) for breast cancer management. For urological cancers this number was 1757 and for lung cancer 677. The Cohort consists of a large repository of routinely collected clinical data recorded both retrospectively and prospectively. The database contains detailed clinical information collected at various timepoints across the treatment pathway inclusive of diagnostic data, and data on disease progression, recurrence and survival.

**Conclusions:**

Guy’s Cancer Cohort provides a valuable infrastructure to answer a wide variety of research questions of a clinical, mechanistic, and supportive care nature. Clinical research using this database will result in improved patient safety and experience. Guy’s Cancer Cohort promotes collaborative research and will accept applications for the release of anonymised datasets for research purposes.

## Background

The burden of disease due to cancer remains substantial. It is the leading cause of death in the UK and it is estimated that one in two people will be diagnosed with cancer at some point during their lifetime. There were around 359,000 new cases of cancer in the UK in 2015, which equates to approximately 990 cases diagnosed every day or about one person every 2 min. Incidence rates for all cancers combined are projected to rise by 2% in the UK between 2014 and 2035, to 742 cases per 100,000 people by 2035 [1].

The new Cancer Centre of Guy’s and St Thomas’ NHS Foundation Trust (GSTT, London) brings together most treatments and research under one roof. The Centre is a place for outstanding cancer care and cutting-edge research, a state-of-the-art facility where patients are diagnosed, prescribed their medication and treated under one roof. As part of our research strategy, we want to invest in pioneering clinical research using real world evidence (RWE).

As a Trust, patient safety and hospital costs remain the most important drivers for accurate data collection and benchmarking, but data from RWE can also create an opportunity for the Trust to play a major role in future decision making and policy changes. An example can be found in the area of prostate cancer. In 2014, the Movember Foundation launched the Global Action Plan Prostate Cancer Active Surveillance initiative (GAP3), which covers the largest centralized prostate cancer active surveillance database to date. Its primary goal is to create a global consensus with uniform guidelines on the selection and monitoring of men with low risk prostate cancer [2]. It includes data on more than 13,000 prostate cancer men on active surveillance from 20 different centres across 12 different countries. The initiative will play a major role in the reassessment of the current guidelines for managing these patients. GSTT contributed data on 500 patients.

Since the value of RWE has also been recognised by regulatory agencies, we established a Research Ethics Committee (REC) approved research database for cancer patients (Reference: 18/NW/0297). Guy’s Cancer Cohort provides a valuable infrastructure to answer a wide variety of research questions of a clinical, mechanistic, and supportive care nature. Clinical research using this database will result in improved patient safety and experience.

The set-up of the Guy’s Cancer Cohort is supported through a variety of project-specific grants.

### Construction and content

Patients are recruited at GSTT, London, UK. All patients over the age of 18 years are eligible following their first visit for a diagnosis of active new or recurrent cancer. Since Guy’s Hospital is a referral centre, Guy’s Cancer Cohort also includes patients from secondary and tertiary hospitals. Currently, Guy’s Cancer Cohort focuses on our three most commonly treated tumour groups: breast, urology, and lung but will be rolled out to include all tumours types in due course. From April 2016 until March 2017, 1388 eligible patients visited GSTT for breast cancer management. For urological cancers this number was 1757 and for lung cancer 677.

Prior to their first appointment, all patients receive a letter or text message containing information about their clinical appointment. This package also contains an Information Governance approved NHS document that explains to patients how their routinely collected clinical data may be used for research in an anonymised fashion. Patients contacted via text message are able to follow a link to an electronic version of the documentation. The document explains the information we hold, how we keep it safe and accurate, and how it supports direct care. It also highlights how this information may support other medical purposes (e.g. medical research) and the patients’ right to object to the use of their information for any purpose other than their direct care. If a patient does want to object, then information on how to do so is provided. However, no patient to date has opted out. We have conducted qualitative research on patient preferences regarding opt out consent in our patient population [3], and hence we expect this number to remain extremely low.

In addition to all prospectively collected routine clinical data, Guy’s Cancer Cohort has ethical approval to utilise all routinely collected anonymised clinical information obtained prior to the date of initiation of Guy’s Cancer Cohort – retrospectively dating back to 2005. Hence, if a person presents with a recurrent cancer, data on the first diagnosis will be available if this occurred in 2005 or later. All retrospective clinical data was routinely collected by the direct clinical care team and only anonymised data is included in the research database. To date, detailed clinical and follow-up information is available in the Guy’s Cancer Cohort for ~ 5150 breast patients and ~ 14,000 urology patients.

#### Follow-up

**Fig. 1:**
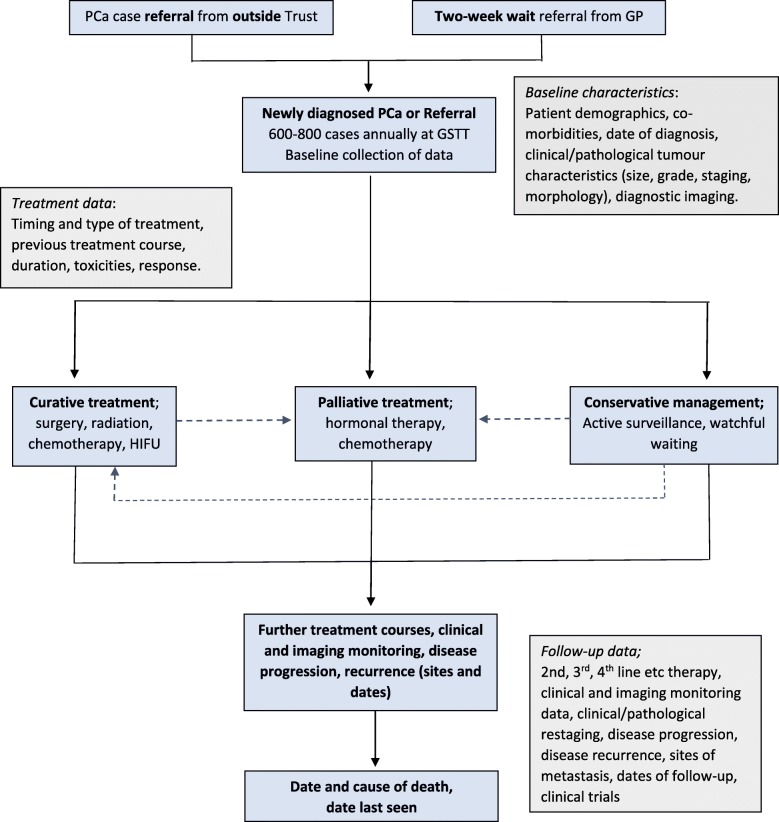
Overview of data collection for men diagnosed with PCa at GSTT

All patients are eligible for participation in Guy’s Cancer Cohort following their first appointment for an active new or recurrent cancer and hence no formal participant assessment will be required. Specific studies conducted using data from Guy’s Cancer Cohort may have more detailed inclusion/exclusion criteria based on the research question asked, but this does not affect patients’ eligibility for the overarching Guy’s Cancer Cohort. Moreover, the accuracy of these criteria will be reviewed and appraised by an Access Committee prior to agreeing to release a specific dataset for research. To illustrate completeness of follow-up, Fig. 1 below provides an overview for men with prostate cancer.

#### Data generation

**Fig. 2:**
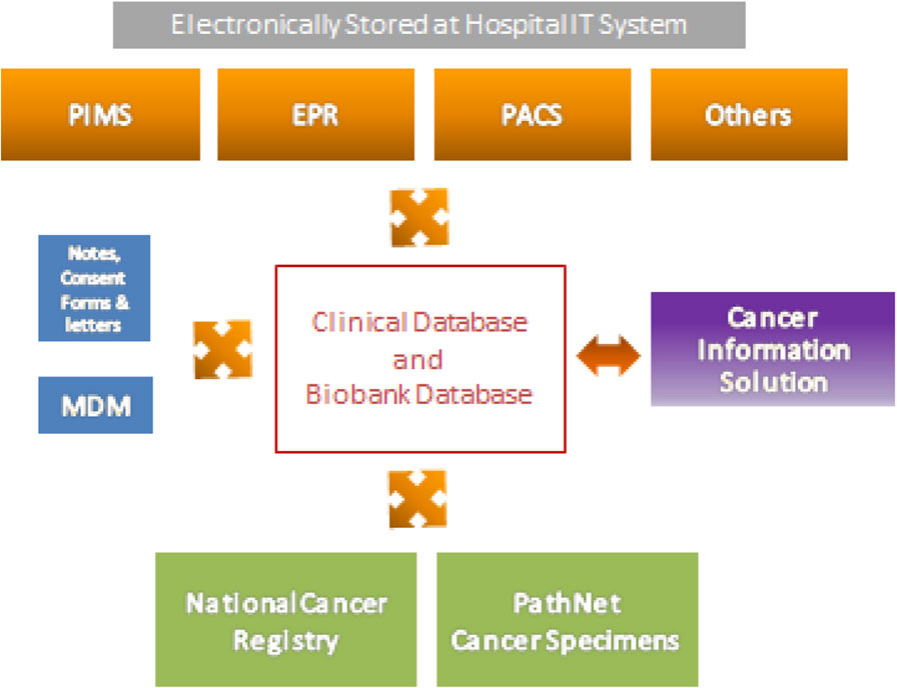
An overview of the different software systems (e.g. Electronic Patient Record (EPR), Multi-Disciplinary Meetings (MDM), Patient Information Management System (PIMS), Picture Archiving and Communication System (PACS)) available at Guy’s to collect patient-specific information

Within Guy’s Cancer cohort, various clinical data is prospectively collected including demographics, tumour characteristics, treatment and imaging data. Clinical data is captured from electronic medical records, referral letters and annual reports for Public Health England (Fig. 2).

Socio-demographic data includes sex, date of birth, age at diagnosis, highest level of education, postal code (to estimate the deprivation index), ethnicity, body mass index (BMI), WHO performance status, and any other relevant routinely collected anonymised data.

The following tumour characteristics are collected: TNM stage, grade, tumour diameter, number of tumours, histology and morphological codes and invasiveness, and any other clinical markers used to define tumour type and severity.

Treatment characteristics comprise data on type and timing of treatment given (e.g. intravesical instillations, systemic chemotherapy, radical cystectomy, radiotherapy or other treatments). For surgical patients specifically, we also collect the following pre-, peri- and postoperative data (Table 1).

**Table 1:** Overview of preoperative, perioperative, and postoperative data collected in Guy’s Cancer Cohort

Preoperative	TNM stage, weight, height, BMI, American Society of Anesthesiologists (ASA) score, previous surgery, radiation or neoadjuvant chemotherapy
Perioperative	Type of surgery, type of lymphadenectomy, blood loss, duration of surgery, accidental organ injury during surgery
Postoperative	Complications, re-operations and re-admissions within 90 days, length of hospital stay, pT stage, number of excised lymph nodes and number of excised and metastatic lymph nodes

Information on other comorbidities (as well as potential treatments) are also recorded as part of standard care using the Electronic Patient Records, provided their relevancy for informing cancer treatment choices.

Information on disease progression, recurrence and survival is collected annually by means of data linkages with Hospital Episode Statistics (HES), the Office for National Statistics (ONS) and electronic patient records.

An overview of the different software systems (e.g. Electronic Patient Record (EPR), Multi-Disciplinary Meetings (MDM), Patient Information Management System (PIMS), Picture Archiving and Communication System (PACS)) available at GSTT to collect patient-specific information is shown in Fig. 1. The routinely collected clinical data held within these various software systems is extracted by a centralised GSTT cancer data team on a monthly basis for inclusion in the compulsory systemic anti-cancer therapy (SACT) and cancer outcomes and services dataset (COSD) national audits. The routinely collected clinical data included in these audits also feed directly into the Guy’s Cancer Cohort clinical datasets and information is linked based on the patients’ hospital number and date of birth. Additional tailored data extractions, specific to the requirements of the Cancer Cohort, are automated and extracted directly out from each hospital software system for validation and quality checking before inclusion in the Cohort clinical databases. The quality and accuracy of the routinely collected clinical data is further improved through clinician-led initiatives to enter the data in a predefined structured format at source. Identifiable information (i.e. hospital number, date of birth, and postcode), whether collected retrospectively or prospectively, is removed prior to the release of the data for research purposes.

Tumour specific database managers work collaboratively with the GSTT cancer data team to validate the extracted datasets and link follow-up information (relating to treatments, disease recurrence or progression) with patient baseline clinical data. Additional information on progression, recurrence or survival is requested on an annual basis from Public Health England (PHE), the ONS and HES, and linked with existing patient information using the identifiers mentioned above. Such requests are funded by the Guy’s Cancer Real-World Evidence Strategy and clinical departments to strengthen the quality and accuracy of the clinical data.

## Utility and discussion

Guy’s Cancer Cohort contributes to the amplification of RWE data science capability and allows to work on challenging cancer research questions. It helps accelerate evidence generation through providing observational studies that can generate new hypotheses and support policy makers. Prior to its formal development, a wide variety of studies had already been published using the clinical data of GSTT’s cancer patients (based on individual ethics or audit approval) – including various studies focused on prostate cancer [4–9], breast cancer [10–13] and renal cancer [14]. The below case study shows how data from Guy’s Cancer Cohort was used to address a clinically important question for bladder cancer:

### Case study

*Neutrophil to lymphocyte ratio (NLR) as a predictor of outcomes in patients with urothelial carcinoma treated with immune checkpoint inhibitors* [15]*.*

**Fig. 3:**
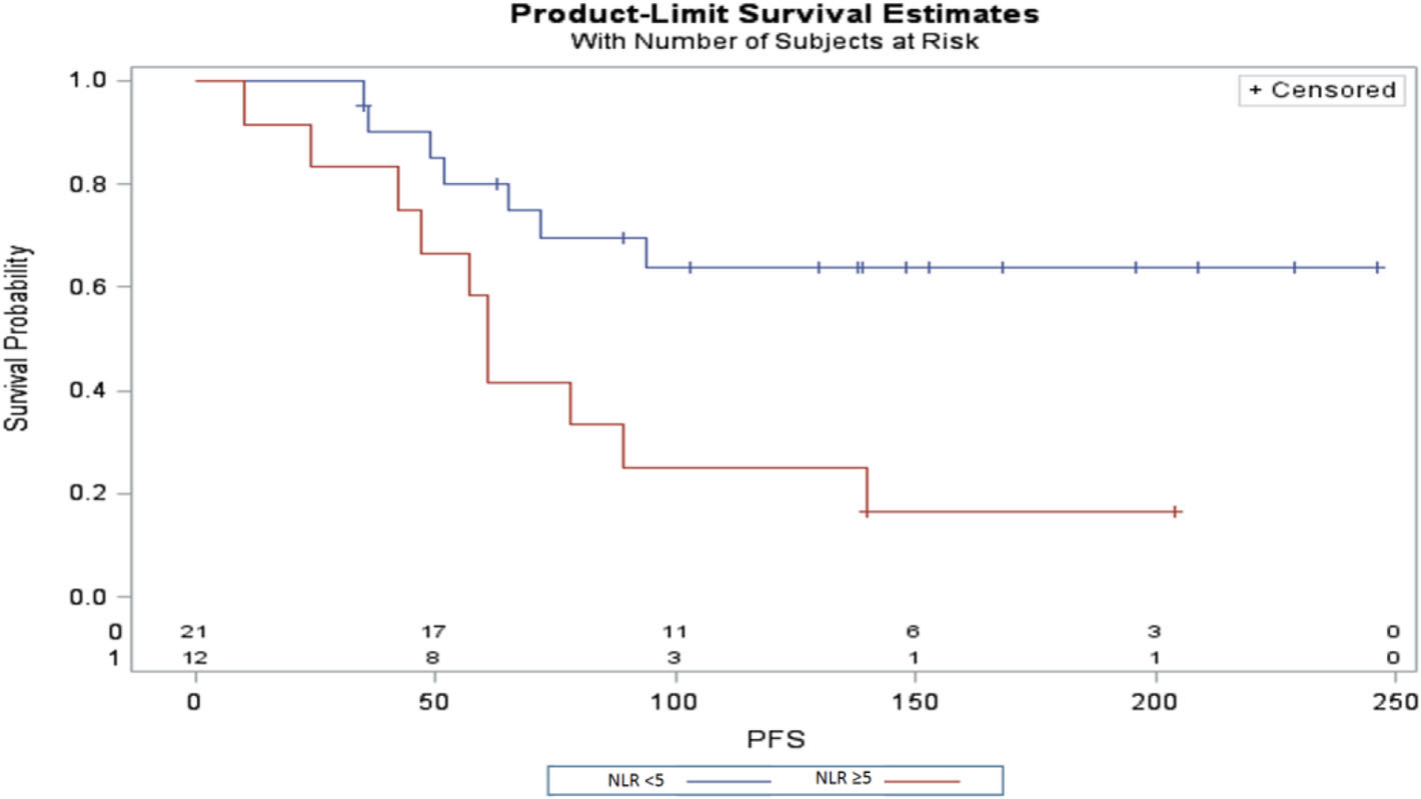
Kaplan Meier Plot for Progression Free Survival for patients who received single-agent pembrolizumab for bladder cancer based on their neutrophil to lymphocyte ratio

This study aimed to determine prognostic factors for the clinical benefit of pembrolizumab in patients with bladder cancer. Thirty-three patients aged 50 to 85 received single-agent pembrolizumab between January and September 2018. When adjusted for age and line of treatment, the hazards ratio for overall survival with NLR > 5 compared to NLR < 5 was 0.11 (95%CI 0.03–0.36). Progression-free survival was also better for those patients with a NLR > 5 (Fig. 3).

### Strengths and limitations

In 2015, the Decision Support Unit of the National Institute for health and Care Excellence (NICE) published a first document highlighting the importance of observational data in clinical decision making [16]. Moreover, the reforms to the Cancer Drugs Fund highlighted the role that real-world data can play in technology appraisals. This change occurred as it has been shown repeatedly that patients in randomised clinical trials (RCT) are highly selected and have a lower risk profile than real-world populations, with the frequent exclusion of elderly patients and patients with co-morbidities [17–20]. Supplementing RCT evidence with data generated from observational settings (e.g. hospitals) can also improve the external validity of oncology drug trials such that physicians treating patients in real-world settings have the appropriate evidence on which to base their clinical decisions [17, 19, 20].

The guidelines from the European GetReal consortium (“incorporating real-life data into drug development”) specifically recommend considering evidence from pragmatic trials and non-randomised studies to improve applicability of treatment effect estimates, inform disconnected or scarce networks of evidence, identify patient populations that will likely receive the drug after launch, and to improve relevant to decision/policy makers and patients [21].

Hence, Guy’s Cancer Cohort has the potential to provide real world data covering the clinical management a wide variety of cancers. Equally, due to the geographical location of the Trust, the data reflects ethnic diversity with about 37% of its population being of black or minor ethnic groups [22]. This provides an opportunity to investigate the racial and ethnic disparities in cancer care, diagnostics, and response to treatment [23]. As with all routinely collected clinical data, there is always the potential for missing data. However, due to the set-up of the information governance of Guy’s Cancer Cohort, a variety of data improvement strategies have been put in place to both prospectively and retrospectively update the clinical data collected.

### Access to the data

Each tumour group has a dedicated access committee, which oversee access to data resources within their specific database (tumour-specific). The role of the Committee is to:

Review applications to access the relevant Database, with reference to scientific merit, study design, requestor’s financial resources (i.e. details of grant to fund study), and the relevant database’s resources. Furthermore, the access committee will rate applications with regard to: strategic value to Guy’s Cancer, the collaborative value to Guy’s Cancer, the resources requested, and the impact of the proposed research. They will review requests for all data (pilot and full studies), as well as results from pilot studies (with view to approving continued access), interim study updates (time to be determined for individual studies), and extension of studies (which will also require a pilot study stage).

The Guy’s Cancer Real World Evidence Programme welcomes specific and detailed proposals for new collaborations from any third party. Initial enquiries should be made to Charlotte Moss (Real World Evidence Programme Coordinator): charlotte.moss@gstt.nhs.uk.

## Conclusions

Guy’s Cancer Cohort showcases the importance of Real-World Evidence for improving patient safety and experience, whilst also providing valuable infrastructure to answer clinically relevant observational research hypotheses. Guy’s Cancer Cohort promotes collaborative research and will accept applications for the release of anonymised datasets for research purposes.

## Data Availability

The Guy’s Cancer Real World Evidence Programme welcomes specific and detailed proposals for new collaborations. Initial enquiries should be made to Charlotte Moss (Real World Evidence Programme Coordinator): charlotte.moss@gstt.nhs.uk.

## Abbreviations

ASA: American Society of Anesthesiologists
BMI: Body Mass Index
EPR: Electronic Patient Records
GSTT: Guy’s and St Thomas’ NHS Foundation Trust
HES: Hospital Episode Statistics
MDM: Multidisciplinary Team Meetings
NICE: National Institute for Health and Care Excellence
NLR: Neutrophile Lymphocyte Ratio
ONS: Office for National Statistics
PACS: Picture Archiving and Communication System
PIMS: Patient Information Management System
RCT: Randomised Controlled Trial
REC: Research Ethics Committee
RWE: Real World Evidence
TNM: Tumour, Node, Metastasis
UK: United Kingdom
